# Evaluating the role of breastfeeding peer supporters’ intervention on the inpatient management of malnourished infants under 6 months in Kenyan public hospitals

**DOI:** 10.1186/s13006-022-00520-6

**Published:** 2022-11-24

**Authors:** Martha Mwangome, Nicole Feune de Colombi, Sophie Chabeda, Edward Mumbo, Julie Jemutai, Benjamin Tsofa, Jacinta Nzinga, Caroline Jones

**Affiliations:** 1 Centre for Geographic Medicine (Coast), Kenya Medical Research Institute/Wellcome Trust Research Programme, P.O. Box 230, Kilifi, 80108 Kenya; 2 Centre for Tropical Medicine and Global Health, Nuffield Department of Medicine Research Building, University of Oxford, Old Road Campus, Roosevelt Drive, Oxford, OX3 7FZ UK; 3 International Centre for Reproductive Health, P.O. Box 91109, Mombasa, 80103 Kenya; 4 Ministry of Health, Kwale County, P.O Box 4, Kwale, 80403 Kenya

**Keywords:** Infants under six months, Exclusive breastfeeding, Malnutrition, Breastfeeding peer supporters, Inpatient records

## Abstract

**Background:**

The 2013 WHO guidelines for nutritional rehabilitation of malnourished infants under six months (u6m) focus on inpatient re-establishment of exclusive breastfeeding and recommends discharge when infant is gaining weight on breastmilk alone. Guided by a breastfeeding support tool, breastfeeding peer supporters (BFPS) can support implementation of these guideline by providing continuous individualised breastfeeding counselling to mothers of malnourished infants u6m. Recording and sharing information plays an important role in shaping in-patient care but little is known about recording practices for inpatient nutrition rehabilitation of infants u6m or how such practices affect care. We set out to explore introduction of BFPS into hospitals, and how it shaped the recording and practices of care for acutely malnourished infants u6m.

**Methods:**

We applied a descriptive, exploratory design involving a pre and during intervention audit of the infant u6m inpatient records in two hospitals in Kenya, as well as pre- and post-intervention in-depth interviews with health workers involved in the care of admitted infants u6m. We developed an audit tool and used it to extract routine data on patient information from hospital records. Data were entered into a REDCap database and analyzed using STATA 17.0 software. We conducted thirty in-depth interviews with health workers exploring their care practices and their perceived effect of the presence of the BFPS on health workers treatment practices. We analysed interview data using thematic framework approach.

**Results:**

A total of 170 and 65 inpatient files were available for the audit during the pre- and post-intervention period respectively. The presence of the BFPS seemed to have encouraged the recording of (i) breastfeeding status upon admission, (ii) breastfeeding management plan and (iii) reporting of its implementation and progress during treatment. The breastfeeding peer support intervention had a positive impact on breastfeeding recording and reporting practices. Health workers reported that the BFPS facilitated the recording of observed breastfeeding data and how their records influenced final inputs of breastfeeding support provided in the inpatient file.

**Conclusions:**

Guideline implementation tools facilitate effective application of guidelines and should accompany any guideline formulation process and have their effectiveness at recording and monitoring progress evaluated.

**Supplementary Information:**

The online version contains supplementary material available at 10.1186/s13006-022-00520-6.

## Background

Globally, acute malnutrition amongst infants aged under six months (u6m) is a major public health problem, with 8.5 million infants u6m suffering from acute malnutrition [1], placing them at significantly greater risk of death from co-morbidities than non-malnourished infants [2]. Sub-optimal breastfeeding feeding among malnourished infants u6m is very common. The prevalence of lactation failure among such infants is up to 90% [3, 4]. The benefits of breastfeeding are widely recognized, and studies suggest that optimizing breastfeeding is particularly important for the survival of malnourished infants recovering from co-morbidities [5, 6].

Exclusive breastfeeding can be successfully re-established among mothers of infants u6m experiencing lactation failure [4, 7] and the 2013 WHO guidelines for nutritional rehabilitation of hospitalised malnourished infants u6m focus on inpatient re-establishment of exclusive breastfeeding [8]. The guidelines also outline recommendations for the assessment of the nutritional status of the infant upon admission, including assessment of observed breastfeeding status, recommendations on supplementary feeds that may be given where breastfeeding is not sufficient or not possible, and assessment of maternal physical and mental health status. They also describe the criteria for discharge. In most low-income settings the 2013 WHO recommendations have been adopted and included in local guidelines, but these are rarely consistently applied [2]. In these settings, challenges such as shortages of health workers, little information on how to effectively re-establish exclusive breastfeeding and a lack of implementation tools to record and monitor the treatment process, all contribute to impeding the effective implementation of the guidelines [9]. Furthermore, the primary cause of admission for many of the hospitalised acutely malnourished infants is infection, with the clinical focus being solely on the treatment of the infection [10]. Consequently, initial breastfeeding and nutrition assessments are often inadequate, little breastfeeding support is provided to mothers during their hospital stay and there is reliance instead on the use of Dilute F100 supplemental milk with many infants ending up being discharged while still on mixed feeding [4].

There is paucity of literature describing inpatient experiences of managing malnutrition in infants u6m.There is even less information on the role of data in nutrition management to inform burden of malnutrition, allocate resources and monitor changes improvement of infants during management. The 2013 WHO guidelines were mainly informed by studies conducted in emergency settings and reported partial success in re-establishing exclusive breastfeeding in an inpatient setting [3, 11]. In 2016, we undertook a study Improving Breastfeeding Among Malnourished Infants (IBAMI) investigating the feasibility and impact on growth of the effective implementation of the 2013 WHO infant nutritional rehabilitation guidelines. The study was conducted among acutely malnourished infants u6m in a public hospital setting in Kenya and, for the first time in a low- and middle-income country (LMIC), we employed breastfeeding peer supporters (BFPS) in a hospital setting to facilitate the process [12]. The BFPS were given specific training in re-lactation standard operating procedures (SOPs), with defined roles and responsibilities including the use of a recording tool aimed at improving documentation and guiding adherence to the guidelines and re-lactation SOP [12]. Within the context of the trial, the health workers in the study reported that the presence of the BFPSs changed the way infant nutritional rehabilitation was managed. Specifically, the presence of the BFPS lead to increasing efforts at re-lactation and decreasing reliance on supplemental milk. BFPSs were said to help address staff shortages and had dedicated time to support and assist the mothers [10].

The IBAMI study was undertaken under trial conditions where research staff were monitoring the activities and recording practices of the BFPS and nurses implementing the nutritional rehabilitation recommendations.

In this paper, we report on a follow-up study developed to understand how BFPS might influence the management of acutely malnourished infants u6m in a routine public hospital setting in Kenya. The study was a descriptive study designed to explore the effect of the BFPS intervention on the inpatient management of malnourished infants under six months in two Kenyan public hospitals. The aim was to identify how the employment of BFPS into the hospitals shaped the recording and practices of care by health workers for acutely malnourished infants.

## Methods

### Study design

The study adopted a descriptive, exploratory approach involving two record audits of the infant u6m inpatient records in each hospital. One before the introduction of the BFPS (pre intervention) and the second during the intervention. The study also involved a pre- and post-intervention in-depth interviews with health workers (nurses, nutritionists and medical doctors) who were involved in the care of the sick infants.

### Study setting

This study was conducted in two public hospitals located in Kenyan rural counties. The two hospitals were selected for the accessibility and availability of a pediatric inpatient admission facility. Though similar in geographic setting, the two were of different levels and capacity. Hospital 1 was a county referral hospital (level 5) offering holistic outpatient and inpatient services with a pediatric bed capacity of 36, while Hospital 2 was a county hospital (level 4) offering both outpatient and inpatient services but with a much lower bed capacity of twelve. A summary of the hospitals’ structure and staffing is provided in Table 1.

**Table 1:** Characteristics of the two study hospitals

Hospital Code	Hospital 1	Hospital 2
Paediatric capacity	36	12
Number of beds	36	12
Monthly admissions	136	26
Paediatric ward staffing	10	11
Paediatricians	1	1
Medical officers	2	1
Clinical officers	0	0
Nurses	6	8
Nutritionists	1	1
Catchment population	23,307	10,201

### Study procedure

1. Malnutrition Guideline recommendations

**Fig. 1:**
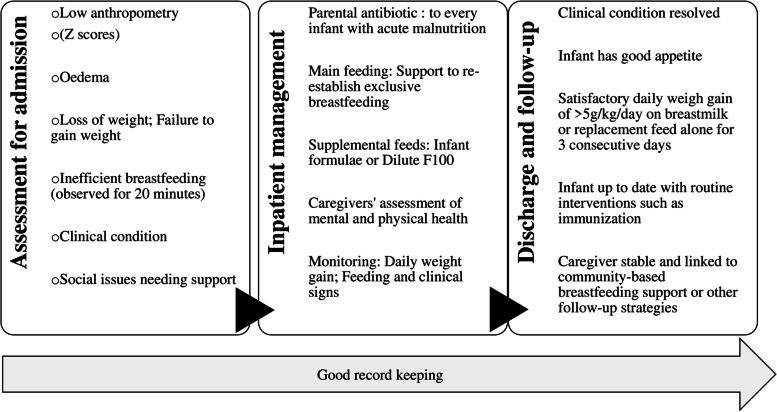
Sequence of events from admission to follow-up of malnourished infants under 6 months

Both the 2013 WHO and 2019 updated Kenyan guidelines for management and treatment of malnutrition among infants u6m recommend a series of activities be undertaken and recorded at admission, during hospitalization and at discharge (Fig. 1). At admission, anthropometry, breastfeeding and clinical evaluation are used to determine nutritional status and severity of disease of the infant. This information feeds onto the management plan dominated by feeding therapy and administration of antibiotics. Discharge is also predominantly defined by anthropometry and clinical improvements. Within the updated guidelines, there is also a recognition of the social and maternal underlying factors and the need to incorporate these into the management plan.

2. Intervention: BFPS and the breastfeeding support tool

The process of recruiting, training and supervising BFPS in this study followed a similar process to what had previously been applied in the IBAMI study [12]. In summary, recruitment was initiated through an advertisement within local community health forums and selection was through an interview process. In this study, two BFPS were recruited, trained and integrated into the health system and posted to work within the paediatric ward of each hospital. The recruitment, grading, day-to-day management and supervision, and remuneration was done by the county government through the respective hospitals, while the study only provided funding for this process. Their role was to work directly under the paediatric ward nutritionists to support breastfeeding of infants u6m admitted to the paediatric ward. The nutritionist led the initial assessments of the mother-infant dyad and together with the BFPS developed the individualised nutritional rehabilitation plan. The nutritionist retained the responsibility to record information in the official inpatient file. The BFPS were provided with a breastfeeding support tool (Additional file 1) to record daily breastfeeding support activities undertaken during infant’s admission. This tool is a 4-page document developed by the study investigators, first applied in the IBAMI study and reviewed for application in this study. It has different sections aimed at assisting the BFPS to provide details of the breastfeeding support activities offered, and document progress for individual mothers-infant dyads. The breastfeeding support tool contained information on infant’s demographic characteristics such as age and admission weight and length. In addition, the tool also allowed for the recording of information on breastfeeding history, a summary of identified challenges and a list of potential remedies. One page of the breastfeeding support tool contains the breastfeeding observation assessment tool (breastfeeding job aid). This is a one page WHO approved tool for guiding health workers to conduct a detailed breastfeeding assessment for all infants. In this study, the job aid was employed by the BFPS once the mother-infant dyad was admitted and settled on the ward. It was used to provide a deeper understanding of the challenges faced by the mother and to identify and discuss new approaches to addressing the challenges. A recording log was also available for the BFPS to record their day-to-day activities and assist in handing over patients should that ever be necessary. In addition, the BFPS tool also contained a weight chart to record daily weight gain of the infants and plot them on a graph for visual representation.

Every morning, before the clinical ward round, the BFPS would conduct weight and breastfeeding assessments, and record progress and new findings in their breastfeeding support tool. The BFPS would then share these records with the ward nutritionist who would use this information to evaluate the feeding plan for each infant and later share progress and recommended changes to the clinical team during the ward round.

3. Data collection methods

- *The record audits – recorded practices of care*

An initial audit of the in-patient records for the twelve months (Jan-Dec 2018) prior to the introduction of the BFPS was conducted at the start of the study in February 2019. A second audit of records was conducted at the end of the study covering the latter 6 of the 9 months (June-Dec 2019) when the BFPS were working in the ward.

Across both the study hospitals, upon admission every patient receives an admission number and has an *inpatient file* opened in their name and admission number. The inpatient file is used to store clinical information of the patient throughout their admission period. It is an open and live document which is updated daily by the clinician, nutritionist and nurses with information on diagnosis, treatment including type of medication, dosage and laboratory tests and results, special feeds etc.

This information is also recorded in two separate records that remain on the ward. The first, the *Inpatient MOH Register,* is a manual register located in the ward and used to record general demographic information, date of admission and discharge as well as diagnose on the patient. The second, nutritional records (recordings of feeding and anthropometry) are kept by the ward-based nutritionists and used to monitor the nutritional progress of the patient. Upon death or discharge of the patient, the inpatient file is closed and taken for storage in the hospital archives whereas the inpatient MoH register and the nutrition records remain in the ward.

An audit tool, informed by the WHO nutritional management guidelines [8], was developed and used to extract data from archived inpatient files (Additional file 2). The tool was developed to follow the sequence of events as illustrated in Fig. 1. It identifies all measurable nutrition and clinical indicators related to care of malnourished infants u6m for each stage of inpatient management process.

The descriptive audit of the hospital records focused on i) providing information on what was being recorded, ii) giving a sense of what may have been ‘missing’ in the admission and care pathway for malnourished infants, and iii) describing the prevalence of malnutrition among infants u6m within the two hospitals.

A total of 192 inpatient files of infants u6m admitted in both hospitals in the pre-intervention period were retrieved from the archives. Of these, one file was blank while 21 inpatient files had information about age that did not match the age information in the inpatient register (age mismatch). As age could not be verified, these 22 were removed from the dataset leaving 170 inpatient files available for audit.

For the post-intervention period, a total of 76 inpatient files of infants u6m admitted in both hospitals were retrieved from the archives. Of these, 11 had an age mismatch and were excluded from the data audit. A total of 65 inpatient files were included in the data extraction activity.

In both pre-intervention and intervention periods, more boys than girls were admitted to each hospital.

Infants were slightly older during pre-intervention period with median age of 30 days (IQR 4 to 90 days) compared to during the intervention when the median age was 21 days (IQR 7 to 90 days). The median hospital stay of six days did not vary between the pre-intervention and intervention periods.

b. *In-depth interviews – perceptions and reported practices of care*

Pre- and post-intervention in-depth interviews were conducted with staff involved in the care of the inpatient malnourished infants u6m to explore their reported care practices, perceptions of how malnourished infants u6m should be managed, and their view on the effects of the presence of the BFPS on these perceptions and practices.

A total of 30 in-depth interviews were conducted with hospital staff: 14 pre-intervention (7 in each hospital) and 16 towards the end of the intervention period (8 in each hospital). Summary descriptions of the participants are shown in Table 2.

**Table 2:** Description of health workers interviewed per hospital

Health workers	Pre-intervention period		During-intervention period	
	Hospital 1	Hospital 2	Hospital 1	Hospital 2
Nurses	5	3	3	4
Medical Officers	0	2	2	0
Nutritionists	2	2	2	2
BFPS	0	0	1	1
**Total**	**7**	**7**	**8**	**8**

4. Sampling and data collection

In the audit, we included files for infants aged below 6 months who had been admitted in either of the 2 hospitals during the period of the first audit (Jan-Dec 2018) and period of the second audit (June-Dec 2019) and whose records were available for the audit.

**Fig. 2:**
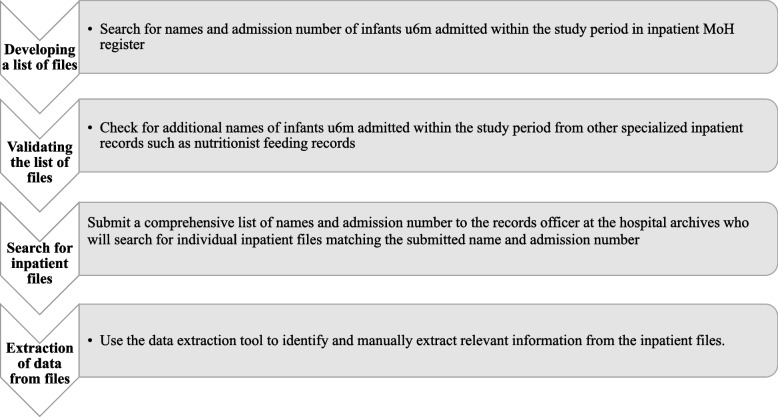
Search process for audit records

The process of finding the records and applying the audit tool involved multiple steps. The key steps involved: i) developing a list of names; ii) validating the list of names; iii) searching for inpatient files and; iv) extracting data from files (Fig. 2).

Each *Inpatient File* located in the records office contained a series of sub-files that had been filled by various cadres of health workers (HWs) during the infant’s admission. Most of the data for the audit were collected from among these various sub-files, but nutrition-specific data (e.g., anthropometry and feeding indications) were also collected from the *Inpatient nutrition Register* (kept by the nutritionist in the wards).

5. Data management and analysis

The extraction and analysis of the audit data involved several stages. First, using the audit tool, data were manually extracted from the archived inpatient files. Files retrieved for the record audit were anonymised and only identified using a study number. In this way, extracted data could not directly be linked to the participant. Data extracted from the files included date of admission, date of discharge, infant demographics including age, gender, anthropometry (weight and length) at admission and discharge, reasons for admission, breastfeeding status at admission and discharge, treatment plan including feeding specifically breastfeeding support and supplementary feeding plans, information on caregiver’s health assessment and treatment, discharge and follow-up information.

The extracted data were double entered into a REDCap database and subsequently downloaded into STATA 15 software (Stata corp. Tx, USA.) for final analysis. Descriptive analysis included the calculations of proportions and medians with interquartile ranges (IQRS) for the variables of interest such as sex, age, weight, length, nutritional status and the availability of nutritional treatment/ management plans.

Interviews were downloaded to a password protected computer and the audio recordings were deleted from the recorder. The audio files were transcribed verbatim and where necessary translated to English. All names, identifiers were removed and replaced with codes. All transcripts were checked by SC against the audio for accuracy, completeness and correctness before they were analyzed using the thematic framework approach. The themes were derived from the three-step sequence of events described in Fig. 1 (assessment and admission, inpatient management, discharge and follow-up) and sub-themes were allowed to emerge from the data. The results were used to complement findings from the record audit.

### Ethical considerations

The study received ethical approval from the Kenya Medical Research Institute (KEMRI) Scientific and Ethics Review Unit (KEMRI/SERU/CGMRC-C/102/3545).

## Results

The main results have been presented following the sequence of events from inpatient admission to post-discharge follow-up of malnourished infants u6m as illustrated in Fig. 1. Both the quantitative and qualitative data is reported using the three key areas; i) assessment and admission, ii) inpatient management, iii) discharge and follow-up, each with appropriate sub-headings to provide further clarity.

### Challenges in data collection

Locating the data required to compare the guideline recommendations with the recorded practices in both hospitals was challenging. There was no specific area in the *Inpatient File* allocated for the recording of the diagnosis and nutritional management of infants u6m (data required for guideline implementation)). A nurse in one of the hospitals explained this:

**Table:** 

*IDI01M* *Okay the nurses we normally record in the cardex, though initially we had incorporated into the file a document that was capturing that* [diagnosis and nutritional management] *just a document for nutrition issues, but then you know when the County came in they started redesigning the files again and that document that we had incorporated was actually removed so we only document in the cardex and then the MO interns also when they review the patients, they also document in their notes.*	

In both hospitals, the same information was sometimes recorded by two different HWs in two different locations of the *Inpatient File*. Such practices sometimes led to different information being recorded for the same infant with some contradictory results (see details under nutritional classification sub-heading).

### Patient’s description

From the patient files reviewed, infants were slightly older during pre-intervention period with median age of 30 days (IQR 4 to 90 days) compared to during the intervention when the median age was 21 days (IQR 7 to 90 days). The median hospital stay of six days did not vary between the pre-intervention and intervention periods.

1. **Infant nutritional assessment, classification and diagnosis at admission**

- *Anthropometric assessment*

In both the pre-intervention and intervention periods, more admission weight measurements were recorded compared to admission length measurements. In the pre-intervention period, admission weight was recorded for 142/170 (84%) of infants while length was recorded for 111/170 (65%) infants u6m. During the intervention period, admission weight was recorded in 47/65 (72%) of admitted infants, length in less than half 30/65 (46%) of the admitted infants u6m.

b) *Nutritional classification*

In the pre-intervention period, 35/170 (21%) of the admitted infants records indicated admission diagnosis as acute malnutrition although an additional 11 infants who had a Z score of less than minus 2 at admission did not receive the malnutrition diagnosis. During the intervention period, acute malnutrition was recorded at admission for a slightly larger proportion 18/65 (28%) of the assessed infants. Only four infants with qualifying Z scores were not indicated as malnourished. It is difficult to make any assumptions about why there might have been this slight increase in proportion of infants with malnutrition diagnosis and it’s particularly puzzling in light of the decline in the proportion of infants who had anthropometry recorded on admission. The sample size is small and the period during the intervention lasted only 6 months (compared to the full 12 months for the pre-intervention audit). It is possible that the intervention was being implemented during a particularly challenging nutrition season. Importantly, it became apparent during the audit that the diagnosis of acute malnutrition could be a contested issue. That is, the diagnosis of acute malnutrition recorded in the inpatient file by the clinician sometimes differed from the recorded diagnosis in the nutritionist’s records. For example, in the pre-intervention audit of data from Hospital 1 the nutritionist’s records reported 32 (25%) cases of acute malnutrition at admission while clinicians reported only 20 (16%) of the same infants as acutely malnourished.

The data from interviews with nurses and nutritionists suggests that one reason for these discrepancies could be that some medical interns either didn’t weigh the infant, or they recorded incorrect weights.

**Table:** 

*IDI05M* *Respondent:*. *.. If it’s the weight, sometimes you can find the clinician maybe is tired he/she will estimate that weight but when we come here we just do it physically, we want to measure the weight we see how is it exactly, the height so that we can get the right measurement for the feedings*	
*IDI05M* *Respondent: So, we will have to redo and see the calculation if they are okay, we will have to re-ask the nutritionist if this formula is okay, you see, so you can’t just rely on what they (MO interns) have said.*. *.*	
*IDI03M* *Respondent: I have gotten that incident like in three times, the MO interns, they change the weights. .. yeah, so that’s the problem. So, I document it in the register so that I may tell them I did it and I got this, so we try to stick with the nutritionist weight, yeah.*	

This situation was exacerbated by the lack of sufficient weighting scale, with scales being moved between wards so that time could be wasted trying to locate the necessary equipment. A nutritionist in Hospital 1 also expressed concerns that the scales were manual and these took considerably longer to use than a digital scale:

**Table:** 

*IDI02M* *Respondent: Another thing are the resources, like in the Paediatric ward we work with the manual weighing scales, we have that one which you have to push it, yeah, so for that, we prefer the digital scale because when you go there in the morning, let’s say we have 15 children that you have to review and you have the manual weighing scale, so for a child you can take five minutes because in the morning you have to reset it again [. ..*] *for you to weigh the child, so we prefer the digital because they are giving accurate measurements. We used to borrow from New Born Unit (NBU) then, for the side of infection there is no need*	

A second possible reason provided by the nurses was that the medical interns did not always correctly calculate the Z score and so were unable to correctly diagnose malnutrition, with their calculations needing to be double checked by the nutritionist.

**Table:** 

*IDI05M* *Respondent: “So we will have to redo and see the calculation if they are okay, we will have to re-ask the nutritionist if this formula is okay, you see, so you can’t just rely on what they (MO interns) have said.”*

c) *Breastfeeding assessment*

In the pre-intervention period, 157/170 (92%) infants had breastfeeding information recorded in their inpatient file. Of the 157 infants, 96 (61%) were reported to be exclusively breastfeeding at admission. However, of the 35 infants diagnosed with acute malnutrition, only 28 (80%) had breastfeeding status recorded at admission, and this was primarily through self-reporting, not by observation as recommended by the guidelines. One of the nurses described that the lack of observations was due to shortages of staff:

**Table:** 

*IDI01K* *Respondent: because of the shortage of staffs, sometimes you find that even if they give it, they don’t give it as a whole, maybe someone will just a mother, are you breastfeeding? are you practicing exclusive breastfeeding? If she says yes, they don’t give further information. .. .*	

In the audit of records from the intervention period, we observed no change in recording of breastfeeding status of infant u6m at admission. While the BFPS were present, a similarly high proportion 59/65 (91%) of admissions had breastfeeding status indicated in their records. Additionally, 43/59 (73%) infants were recorded as being exclusively breastfeeding at admission. Of the 21 infants diagnosed with acute malnutrition, breastfeeding status was recorded for 19/21 (90%) of them.

d) *Maternal/Caregiver’s health evaluation*

The audit of records from the pre-intervention period, indicated that fewer than a quarter of caregivers 38/170 (22%) received some form of maternal health assessment. By contrast, in the audit of records completed during the intervention almost a half of care givers 29/65 (45%) received some form of physical and mental assessment. Of the 29 caregivers assessed during the intervention implementation, two were recorded as experiencing mental health challenges requiring specialized treatment. However, in neither case was there any record of the maternal mental health assessment tools used in the diagnosis and no information was recorded on the treatment or referral recommended for the caregiver.

Though not recorded, the interview data suggests that the nutritional status of the mothers was also assessed and if a mother was viewed as being undernourished, they were registered into a supplementary nutrition support programme.

**Table:** 

*IDI02M* *Respondent: We counsel them (mothers). .. if it happens that the nutritional status of the mother is not that conducive or convincing, so we have to put the mother into supplementary program to support the mother with nutritional supplements*

2) **Infant nutritional management during admission**

In the pre-intervention record audit, only a fifth (36/170; 21%) of inpatient files contained an infant nutrition management plan. The audit of inpatient files completed during the intervention period revealed a considerable improvement in recording of a nutritional management plan with over four fifths of the infants (55/65; 85%) having a nutritional management plan recorded in their admission file.

From the interviews, we learn that the BFPS worked alongside the nutritionist who used information collected by the BFPS to populate inpatient documents. It seems the nutritionists used information recorded by the BFPS in her tool to populate the nutrition register and inpatient file as illustrated in the quotes below.

**Table:** 

*IDI02M* *Moderator: Alright,. .. you had earlier on mention about some of the changes in practice that you experienced through the breastfeeding peer supporter’s intervention. Was there a change now in the data recorded when you’ve been having the breastfeeding peer supporter?*	
*Respondent: Yeah, there is, there is because like apart from her using the counselling forms in for example in pediatrics, the register, the register is also capturing these young infants who are below 6 months and they’ve been having challenges with breastfeeding and the came with severe acute malnutrition, yeah that was actually being documented on a daily basis.*	
*Moderator: So, she was documenting all that information?*	
*Respondent: The nutritionist were the ones who actually used to document the register, but her she used to have those forms where she actually documents the counselling forms together with those other research forms.*

- *Feeding plan/re-establishing exclusive breastfeeding*

In the pre-intervention period, 104/170 (61%) infants had a detailed breastfeeding management plan recorded. In the records, details were given in reference to demonstrating breastfeeding techniques such as positing and attachment, enrolling mothers into a nutrition support program or prescription of lactogogue to mothers to initiate breastmilk production. Further, among the 35 infants diagnosed with acute malnutrition, there was detailed records indicating that for 21 of these mother’s (71%) their milk was supplemented with formulae (F-100, F75, or Prenatal nan formulae).

Prior to the intervention, the data from the interviews suggested that breastfeeding support was not a central part of the feeding plan for acutely malnourished infants with nutritionists reporting that would give supplemental milk to infant as they “waited” for breastmilk to increase:

**Table:** 

*IDI02K* *Respondent: Okay, when we assess we have said that we assess the mother’s milk by expressing, so when we express two to three times and we don’t attain the quantity that we need the child to feed per maybe after three ours or two, then we know the child is being underfed, so we look for an alternative, as the mother we supplement with either porridge so that we increase the milk, we do have something to support the child as we wait the milk to increase, so we use the F-100 dilute*	

By contrast, in the intervention period, 55/65 (85%) infants had a detailed breastfeeding management plan recorded in their inpatient file. The majority of these plans included details on how to support caregivers improve breastfeeding techniques, and how to support cup feeding of hand expressed breastmilk. Of the 21 infants diagnosed with acute malnutrition, only a third (7/21:33%) were recorded as having received Dilute F100 milk while the majority were recorded to have been supported to retain exclusive breastfeeding.

The interview data suggest that the presence of the BFPS in the ward provided the workforce and time needed to implement the feeding guidelines more effectively reducing overdependence on diluted F100.

**Table:** 

*IDI02 K* *Respondent: Ok. the peer supporter was of great help in the management of malnourished children in the ward especially the under 6 months children coz after admission, we do calculate the feeds but also, we give the time to the peer supporter to at least talk to the mother. So far for the mothers of the children that we had in the ward while we gave the peer supporter time to discuss with the mother, we found that after two to three days we found that the milk the breast milk started flowing very good and we could even stop the formula milk and just continue with the breastfeeding only*	
*IDI02 M* *Respondent: Yeah, of course there was effect because we saw children who actually came in with very low weight actually finally gaining weight and finally getting discharged when the mothers have really established breastfeeding effectively. Yeah, so that was very, very important and like initially, initially it was a bit of a challenge because the first thing that we would ever thought about was any time a mother came in would first think about supplementary feeds that is F100, yeah so, F100 diluted and then see whether we can. .. we used that’s what we used to do and then support the mother with that as we do counselling to see whether they can actually establish breast milk, but when we started then we realized that it was very much easy to achieve not just by using artificial feeds, but just counselling and showing the mother how to attach it was actually working very effectively.*

3) **Infant discharge and follow-up**

- *Discharge*

Both prior to and during the intervention, important discharge information was missing from the records of the infants u6m. In the pre-intervention period, three infants died after admission. Discharge anthropometry was recorded for less than a quarter 39/167 (23%) of the discharged infants with a similar percentage having their breastfeeding status recorded 36/167 (22%). During the intervention period, six infants died after admission. Discharge weight was recorded for 18/59 (31%) and length recorded for 6/59 (10%) of infants. Exclusive breastfeeding at discharge was recorded for 18/59 (31%) while 6/59 (10%) infants were recorded to have been discharged while still on supplemental dilute F100 feed.

The nutritionist in both hospitals suggested that a potential reason for the lack of anthropometric and breastfeeding status data from the discharge records was that they were often not on duty when the discharge process was taking place. The clinical team were in charge of discharge decisions, and it became clear that the primary criteria for discharge was clinical rather than nutritional rehabilitation. This was particularly true when there was pressure for beds. Consequently, infants could be discharged without their nutritional status being adequately assessed and recorded:

**Table:** 

*IDI03M* *Respondent: Now what happens it’s the MO interns, you know like today we were having pressure, they wanted to discharge the patient, they say we have very many patients in the wards, so they say let’s discharge so that we have less patients, so that we may work. So, in a situation like that like today I have held two children under six, I have told them do not discharge until tomorrow we see how this child will be, because since yesterday they have not been adding weight, they didn’t add weight yesterday, today they have not added weight, so I told them let’s wait until tomorrow we see if there is a gain. So, in incident like that for now am here, when I go back maybe they are already discharged, because the MO wants to discharge and am telling him not to discharge, so when I go and most of the time am not in the ward, they discharge, especially with the MO interns.*	

By contrast, the status of clinical conditions of the infants at discharge were recorded in 133/167 (80%) of the infant’s pre-intervention and in 50/59 (85%) of the infants during the intervention.

b) *Follow-up*

The guidelines recommend follow-up after discharge through community-based breastfeeding support programmes or out-patient clinics. In this audit there were no records of the linkage of caregivers to community-based breastfeeding support programmes, either prior to, or during the intervention. During the intervention period, follow-up with breastfeeding support at the hospital level was recorded for 29/59 (49%) of the infants.

In the pre-intervention interviews, nurses and nutritionists expressed concerns about the lack of support for effective post-discharge care and follow-up, reporting that many of the infants just ended up back in hospital:

**Table:** 

IDI05M *Respondent: Yeah, coz the mother. .. maybe after we discharge the mother you give them the right information as per the guidelines but when they go at home, they do their own things, and the baby still comes here with the same, same acute malnutrition, yeah* *Moderator: Does this happen so often?* *Respondent: Yeah, it does, [[I: Mm]] mm, when you ask, they will always tell you ooh, I did give cow’s milk, because I was not able to produce enough, such like things, so it’s challenging, you can’t be able to implement the WHO because of that*	

However, during the intervention when the BFPS were in place, they sometimes took the initiative to follow-up on infants’ post-discharge to check on how they were progressing:

**Table:** 

IDI04K *Moderator: How was this follow up as in when?* *Respondent: Aah what she (BFPS) would do is if like she has identified the place where the clients come from she would even visit them to their homes to see how they are progressing and if not that they would be, they were given a returning date to come to the facility to see 202how they are progressing.*	

This follow-up, together with the higher success rate of relactation, gave the nurses and nutritionists the impression that the intervention decreased the number of days in hospital stay for the infants.

**Table:** 

*IDI03K* *Respondent: First, it showed that when there is proper implementation or maybe a mother is well supported to breastfeed, then. .. we decrease that stay in the hospital.. .*	

## Discussion

We set out to explore the effect of a breastfeeding peer supporter intervention on the inpatient management of malnourished infants under six months in two public hospitals in Kenya. We employed an audit of inpatient records and in-depth interviews with the providers of care as to identify how the addition of BFPS into the hospitals shaped the practices of care for acutely malnourished infants u6m. We found that the BFPS intervention made little difference to how admission and discharge anthropometry; weight and length measurements were recorded. This is not particularly surprising since the BFPS are not involved in the admission or discharge process. What the data did reveal is that different cadres of staff were occasionally liable to classify infants differently due to a range of factors including the lack of adequate equipment (scales) and inadequate training of interns. Clinical interns were more likely to admit and discharge infants based on the status of their clinical condition and not necessarily their nutritional or feeding status. While this did not appear to impact significantly on the management of the infants (the nutritionists basing their management plans on their own anthropometry measures) it is likely to impact on any assessments of the burden of malnutrition that are based on the data contained in the inpatient file.

### Influence of intervention on inpatient records

The paucity of adequate recording of patient data is reportedly common in many LMICs including Kenya [13, 14]. Studies assessing the relationship between the quality of patient records and the occurrence of adverse events have shown that the quality of patient records is a predictor of the quality of care received by the patient. This implies that improving the quality of records may lead to improved clinical care and survival [15]. Efforts to enhance awareness of the burden of acute malnutrition in infants u6m need to include interventions that promote and facilitate effective measurement and recording of the nutritional status of all infants u6m admitted to inpatient care.

Despite the lack of influence on admission recording practices, the presence of the BFPS and their lactation forms, did seem to encourage the recording of breastfeeding status at admission, with the health workers describing how the BFPS also facilitated the recording of observed and not just self-reported breastfeeding data. In addition, the presence of the BFPS encouraged the recording of the breastfeeding management plan and reporting of its implementation and progress during treatment. The BFPS intervention was designed to facilitate the implementation of the infant nutritional rehabilitation guidelines, focusing on re-establishing exclusive breastfeeding and relactation, so it was not surprising that it had most impact on the recording, reporting and implementation of breastfeeding practices. The BFPS developed the breastfeeding support plan alongside the pediatric ward nutritionists and detailed their breastfeeding support activities in their breastfeeding support tool which was reviewed regularly by the ward nutritionist. Even though the BFPS had no authority to record any information in the inpatient files, the close interaction and frequent discussions on the breastfeeding support plan between the BFPS and the ward nutritionists influenced the breastfeeding details that were eventually recorded in the inpatient files. Without the BFPS and their breastfeeding support tool, such information may not have been recorded and the effectiveness of the approach and the need for additional support or other inputs would have been difficult to monitor. Such monitoring and recording is central to effective clinical care [15] and is equally important to the successful reestablishment of exclusive breast feeding and nutritional rehabilitation. Our findings suggest that, to date, this focus on the progress of breastfeeding has been a neglected, primarily due to staff shortages, but also because there are no tools available to monitor and report progress. Data from studies on other aspects of healthcare suggest that activities that fall outside routine reporting requirements are the activities that are most often neglected when staff are over worked [16]. This also supports our findings from the interviews with health workers and BFPS (reported on elsewhere) that the presence of the BFPS provided not only the ‘person time’ to support relactation but also the visible reminder of the importance of nutritional rehabilitation and the possibility of implementing the guidelines [10]. The tool also provided the BPFS with a ‘legitimacy’ and a defined role (10), similar to the way in which the ‘Kardex’ (a handwritten record of treatment is given to in-patients) functions to help define the responsibilities of a nurse [17].

### The value of tools to facilitate guideline implementation

Typically, WHO guidelines are developed with an expectation that countries will adopt them to suit their context and facilitate their implementation including interpretation, developing appropriate implementation algorithms and indicators and training of health workers. The 2013 WHO guidelines for inpatient management of malnourished infants under six months [8] is no different. Before the 2013 guidelines, recording of nutritional rehabilitation for inpatient malnourished infants u6m has long been ignored because of lack of management recommendations. Although the 2013 guideline emphasise the re-initiation of exclusive breastfeeding to infants before discharge, it does not provide tools that may be used to facilitate effectively re-establish breastfeeding in an inpatient setting. However, the presence of the guidelines presents an opportunity to improve on management and provides information on relevant indicators that could be recorded and monitored [8]. Our study has shown that there is potential to improve the guidelines implementation not only by introducing BFPS whose main task is to re-initiate and support exclusive breastfeeding, but also by introducing a breastfeeding support tool to ensure accountability and objective evaluation of workload and implementation process.

The use of tools to facilitate implementation of guidelines is a well-known phenomenon. Research shows that guidelines featuring Guideline Implementation tools (GItools) are more likely to be used than those without GItools [18], however few guidelines offer GItools and guidance on developing GItools is lacking [19]. Generally, GItools work by offering a step-by-step process in implementation and support objective interpretation and implementation of guidelines. Such algorithms can be useful in settings where health workers are overwhelmed with heavy workloads and have to make difficult decisions on prioritizing tasks to complete. Additionally, in this study, the breastfeeding support tool worked to clearly define the role of the BFPS and helped in maintaining boundaries in practice for the BFPS who are not yet a recognised cadre of staff in Kenya [9]. We recommend that guideline developers including WHO should consider providing GItools alongside guidelines to enhance uptake and implementation of these guidelines. But it’s not only about providing tools but making sure that they are integrated into routine hospital practices. This requires facilitative leadership, continued mentorship, resource investment and innovation.

### Influence of intervention on nutritional assessment

The intervention had little or no impact on other important aspects of nutritional rehabilitation of malnourished infants. Of note, is the observed decline in recording anthropometry at admission between pre and during intervention periods; 65 to 46% recording in length. It is not clear what may have caused the decline in recording especially because this has direct implications on diagnosis of acute malnutrition using weight-for-length (WLZ) measure at admission. We know that length measurement is a difficult and least reliably measured among infants u6m [20, 21]. From the interview with the health workers, we know that during the intervention period nutritionists grew to rely on the BFPS tool to collect and record important information on infants. It is therefore possible that nutritionists collected this information but found little value in transferring this information to the inpatient file. Still, it would be important to record anthropometric information in the inpatient file as this is the most public document for all health workers managing the infants and especially because diagnostic cut-offs for other alternative anthropometry like Mid-upper arm circumference (MUAC) [22] are yet to be approved for this age group.

Lastly, the study showed a small proportion of caregivers of infants undergoing some form of health assessment; 22% in pre intervention and 45% in post intervention. The records were unclear on the exact assessment that was conducted, the treatment and or recommendations that were given, including referrals to other departments for further testing and treatment. We know that maternal health assessed by indicators such as body mass index (BMI) or MUAC among others is a good predictor of infants health and nutrition as this may affect the quality and duration of breastfeeding [23]. Hence the goal of any treatment plan for small and nutritionally at-risk infants u6m should be to not only care for the infant but to consider the health and wellbeing of mother-infant dyad for a more sustainable outcome.

### Study limitations

In the intervention period, due to unavoidable circumstances, we were unable to collect data for the full 12 months of intervention. There was also a high rate of missing information in the inpatient file data that was used for the audit. We were also unable to find and interview clinician’s post-intervention and have no views from this cadre on the intervention.

## Conclusions

Data from the record audit and health workers interviews show that breastfeeding peer support intervention had an impact on recording and reporting practices specifically on aspects of the guidelines that focused on supporting mothers to re-establish exclusive breastfeeding. The presences of the BFPS and their breastfeeding support tool played a critical role in ensuring that breastfeeding became central in managing inpatient malnourished infants under six months as recommended by the guidelines. Guideline implementation tools should accompany any guideline formulation process and have their effectiveness at recording and monitoring progress evaluated.

## Supplementary Information

**Additional file 1.** Breastfeeding support tool. A copy of the breastfeeding support tool used by breastfeeding peer supporters to support breastfeeding among study participants

**Additional file 2.** Inpatient record audit tool. A copy of the inpatient record audit tool used by the study research officer to extract data from the archived inpatient files

## Data Availability

The datasets used and/or analysed during the current study are available from the corresponding author on reasonable request.

## Abbreviations

BFPS: Breastfeeding Peer Supporters
BMI: Body Mass Index
GI tools: Guideline Implementation tools
HW: Health Worker
IBAMI: Improving Breastfeeding Among Malnourished Infants
IDI: Individual Interview
IQR: Inter-Quartile Range
LMIC: Low- and Middle-Income Countries
MOH: Ministry of Health
MUAC: Mid-Upper Arm Circumference
U6m: Under 6 months
SOPs: Standard Operating Procedures
WHO: World Health Organization
WLZ: Weight-for-Length Z scores
