# Supplementary material for: Evaluating the role of breastfeeding peer supporters’ intervention on the inpatient management of malnourished infants under 6 months in Kenyan public hospitals
Source: Int Breastfeed J. 2022 Nov 24;17:79. doi: 10.1186/s13006-022-00520-6 (PMC9685898; doi:10.1186/s13006-022-00520-6)
Supplement: Supplementary file 1 — Additional file 1. Breastfeeding support tool. A copy of the breastfeeding support tool used by breastfeeding peer supporters to support breastfeeding among study participants [file 13006_2022_520_MOESM1_ESM.pdf]

BREASTFEEDING SUPPORT FOR ADMITTED NUTRITIONAL VULNARABLE INFANTS AGED BELOW 6 MONTHS

Patient/Participant's Name:___________________Serial/OPD No: ___________ Adm weight(kg):____.____

Date of Birth:_________________Date of Admission: _____________ Age in days:___________________

Birth weight (kg): _____. _______ Birth Place___________________ Residence: ____________________

Direct Admission ☐ Hospital/departmental referral ☐ Discharge on admission ☐

WLZ<-2 ☐ WAZ<-2 ☐ pitting oedema ☐ Failure to gain weight ☐ Recent weight loss ☐

Reason for admission *(Summary of why they are here)*

____

____

____

____

Congenital malformation*: ☐YES, ☐No*

Breastfeeding History (pre-lacteal feeds, mixed feeding, bf frequency, bf at night, is breastmilk enough?)

____

____

____

____

____

____

____

____

Breast assessment (clean, healthy, nipple size and shape, oxytocin reflex, pain, discomfort)

____

____

____

____

____

____

____

____

Breastfeeding Observation (use WHO observation aid overleaf and observe mother, baby, positioning, attachment, suckling)

____

____

____

Main breastfeeding concerns (Tick where appropriate)

Infant

☐Delayed start
☐Infrequent feeds
☐No night feeds
☐Short feeds
☐Poor attachment
☐Poor positioning Tiredness
☐Partial lactation failure
☐Complete lactation failure

Mother

☐Perceived milk insufficiency (not enough milk)
☐Lack of confidence
☐Worry, stress
☐Dislikes breastfeeding
☐Rejection of baby
☐Bottle feeding/pacifiers

Others:

____

____

____

____

Breastfeeding Support package

☐How breastfeeding works
☐Advantages of exclusive breastfeeding *(fore and hind milk)*
☐Breastfeeding technique *(positioning, attachment and Suckling)*
☐Common breastfeeding difficulties *(not enough milk, crying baby)*
☐Assist carer establish good feeding frequency *(night feed)*
☐How to express, store, reheat & cup feed breastmilk *(stimulate oxytocin reflex)*
☐General Hygiene manners
☐Child stimulation (play with baby, massage)
☐Supplemental suckling technique (SST)
☐Treatment of breast condition *(engagement, inverted nipples)*
☐Maternal nutrition
☐Birth spacing

Note: To be completed by nutritionist and Peer supporter

 PS Initials: _____________ *Nutritionist initials:________________*

If the baby is not too sick to suckle, find an opportunity to observe the mother and infant breastfeed. Use the check list below to document your observation

**Signs breastfeeding is going well**	**Signs breastfeeding is not going well**
Mother	
☐ Mother looks healthy	☐ Mother looks ill or depressed
☐ Mother relaxed and comfortable	☐ Mother looks tense and uncomfortable
☐ Signs of bonding between mother and baby	☐ Mother/baby eye contact
Baby	
☐ Baby alert and able to breastfeed	☐ Baby sick and too weak to breastfeed*
☐ Baby calm and relaxed	☐ Baby is restless or crying
☐ Baby roots or reaches out for breast if hungry	☐ Baby does not reach or root
Baby Positioning	
☐ Baby's head and body in line	☐ Baby's neck and head twisted
☐ Baby held close to mothers' body	☐ Baby not held close
☐ Baby's whole body supported	☐ Baby supported by head and neck only
☐ Baby approaches breast nose to nipple	☐ Baby approaches breast, lower lip/chin to nipple
Baby attachment	
☐ More areola seen above baby's top lip	☐ More areola seen below bottom lip
☐ Baby's mouth open wide	☐ Baby's mouth not open wide
☐ Lower lip turned outwards	☐ Lips pointing towards or turned in
☐ Baby's chin touches breast	☐ Baby's chin not touching breast
Suckling	
☐ Slow deep sucks with pauses	☐ Rapid shallow sucks
☐ Cheeks round when suckling	☐ Cheeks pulled in when suckling
☐ Baby releases breast when finished	☐ Mother takes baby off the breast
☐ Mother notices signs of oxytocin reflex	☐ No signs of oxytocin reflex noticed

Observation notes:

____
____
____
____
____
____
____

Peer supporter activities log sheet

Day 0 (admission day)

____

____

____

____

Weight(kg):________, weight gain(gm):_________

Day 1:_____________________________________

____

____

____

____

Weight(kg):_________, weight gain(gm):________

Day 2:_____________________________________

____

____

____

____

Weight(kg):_________, weight gain(gm):________

Day 3: ____________________________________

____

____

____

____

Weight(kg):________, weight gain (gm):_________

Intensive review (nutritionist and peer supporter)

Date:_____________ Nutritionist initials:_______

Weight gain (Steady weight gain?)

____

____

Maternal milk production (Increase in milk production?)

____

____

Recommendations

____

____

____

Day 4:_____________________________________

____

____

____

____

Weight (kg)_______, weight gain (gm):________

Day 5*:*____________________________________

____

____

____

Weight (kg):________, weight gain (gm):________

Day 6*:*____________________________________

____

____

____

Weight (kg):_________, gain (gm):_____________

Day 7*:*____________________________________

____

____

____

____

Weight (kg):__________, gain(gm):______________

Day 8:_____________________________________

____

____

____

____

Weight (kg): __________; gain (gm): ____________

Day 9: ____________________________________

____

____

____

____

Weight (kg):____________, gain (gm):__________

WEIGHT CHART FOR MALNOURISHED INFANTS BELOW 6 MONTHS

Name: __________________________ Age: _____months_____days Birth weight:____kg____g

Weight (KG)

.0																				
.5																				
.0																				
.5																				
.0																				
.5																				
.0																				
0	1	2	3	4	5	6	7	8	9	10	11	12	13	14	15	16	17	18	19	20

Days

Review for discharge: Date:______________ Nutritionist initials: _______Weight:______; Length:_______

☐Good appetite
☐Weight gain on breastmilk only for the last 3 days
☐Oedema resolved

Discharge notes:____
____
____
____
