# Supplementary material for: Evaluating the role of breastfeeding peer supporters’ intervention on the inpatient management of malnourished infants under 6 months in Kenyan public hospitals
Source: Int Breastfeed J. 2022 Nov 24;17:79. doi: 10.1186/s13006-022-00520-6 (PMC9685898; doi:10.1186/s13006-022-00520-6)
Supplement: Supplementary file 2 — Additional file 2. Inpatient record audit tool. A copy of the inpatient record audit tool used by the study research officer to extract data from the archived inpatient files [file 13006_2022_520_MOESM2_ESM.pdf]

RECORDS REVIEW FOR INPATIENT INFANTS WITH SEVERE ACUTE MALNURITION

The aim of this tool is to conduct a review of records and recording practices relating to the nutritional status and nutritional rehabilitation management of inpatient malnourished infants <6months

Hospital code ____ _____

Date of admission ___ ___/__ __/ __ __ __ __

Date of discharge ___ ___/__ __/ __ __ __ __

DEMOGRAPHICS

During admission

Gender of the infant

Male ☐ Length __ __ Z score __ __

Female ☐ Weight __ __ Weight at Birth ____

Age of infant Months _____ Weeks ______ Days _____________

ADMISSION

Reason for Admission

Infection ☐

Specify ____________________________

____

____

Recent weight Loss ☐

Poor weight gain ☐

Pitting oedema ☐

Low Z score ☐

General danger sign(s)

Unable to drink or breastfeed ☐

Vomits everything ☐

Prolonged convulsions > 15mins ☐

Lethargy ☐

Unconscious ☐

Currently Convulsing ☐

Medical or social issues of the mother

1. Disability ☐
2. Depression of caregiver ☐
3. Other adverse social circumstances ☐
   Specify ____

Any documentation to support ____

Prescribed Treatment for the infant during admission

Antibiotics prescribed Yes ☐ No ☐

1. ____________________________________
2. ____________________________________
3. ____________________________________

Other Medical conditions treatment:

Other Medical Conditions Yes ☐ No ☐ (If yes, continue to 1-4 below, if No, move to the feeding methods section)

1) TB Yes ☐ No ☐

Name of the medicine____

2) HIV Yes ☐ No ☐

Name of the medicine____

3) Surgical Condition Yes ☐ No ☐

Any supporting documentation ____

4) Disability (specify disability i.e congenital heart disease, cerebral palsy,
   Yes ☐ No ☐

Any supporting assessment tool ____

FEEDING METHODS

Is the infant breastfeeding status recorded? Yes ☐ No ☐

What is the recorded breastfeeding status? Any breastfeeding, exclusive, predominant, partial, none,
Any documentation to support assessment of breastfeeding status____________________
Does the infant have any breastfeeding concerns (directly observed for 15- 20min, in a supervised separated area) (select all that apply)

1. Positioning [ ]
2. Attachment [ ]
3. Suckling [ ]

Others (specify) ____

Any documentation to support ____
Is there a nutritional management plan?
Yes [ ] No [ ] No data available [ ]
Summarise details of the plan ____
If breastfeeding, has the mother or care giver supported to retain or re-establish exclusive breastfeeding? Yes [ ] No [ ]
Summarise details of the support given____
If the infant is not breastfeeding, has the mother or caregiver been supported to re-lactate?
Yes [ ]
No [ ]
Any details of the support given____
Is wet nursing practiced Yes [ ] No [ ]
Comment____

Supplementary feeds during admission

1) If Infant had SAM with no oedema:

Is the infant given expressed breast milk? Yes [ ] No [ ]
Is the infant fed on commercial Infant formula? Yes [ ] No [ ]
Is the infant fed on F-75 or dilute F-100? Yes [ ] No [ ]

2) If infant has SAM with oedema:

Is the infant fed on expressed breast milk and or infant formula or F-75?
Yes [ ] No [ ]

Mothers or caregiver's health during admission

Was the mother or caregiver physical and mental health assessed during admission?
Physical health Yes [ ] No [ ]
Details of the assessment____
Mental Health Yes [ ] No [ ]
Details of assessment ____
Was the mother offered treatment or support? Yes [ ] No [ ]
Details of support offered____

DISCHARGE

Checking to see if the following information is recorded/indicated
Weight ______________ Length __________ Z Score ___________
Is the infant clinically well and alert Yes [ ] No [ ]
Comment____
Have the clinical (infections) or medical conditions including oedema resolved
Yes [ ] No [ ] Comment_______________
Was the appetite test done? Yes [ ] No [ ]
Does the Infant have good appetite? Yes [ ] No [ ] Comment_____________
Is the infant exclusively breastfeeding? Yes [ ] No [ ] Comment _____________
Is the infant feeding well with replacement feeds Yes [ ] No [ ] Comment ______
Is the infant gaining weight on breastmilk only Yes [ ] No [ ]
(5g/kg/day for at least 3 successive days) Comment_____________________________
Is the infant gaining weight on replacement feeding only?
Is the weight gain for length ≥ -2 Z-score Yes [ ] No [ ] Comment ____________
Is the infant gaining weight on breastfeeding and replacement feeding?
Yes [ ] No [ ] not indicated [ ]
Was the infant checked for immunization? Yes [ ] No [ ] Comment____________
Was the infant checked for other routine interventions? Yes [ ] No [ ]
Infant feeding in the context of HIV Yes [ ] No [ ] Comment________________
Counselling for Low birth weight Yes [ ] No [ ] Comment_________________
Was the mother or caregiver linked to community-based follow-up and support?
Yes [ ] No [ ] Details: ____
Follow-up breastfeeding counselling support offered at facility level
Yes [ ] No [ ] Details: ____
Follow-up breastfeeding counselling support offered at community level
Yes [ ] No [ ] Detail: ____

Comment____
